# ZEB2, the Mowat-Wilson Syndrome Transcription Factor: Confirmations, Novel Functions, and Continuing Surprises

**DOI:** 10.3390/genes12071037

**Published:** 2021-07-03

**Authors:** Judith C. Birkhoff, Danny Huylebroeck, Andrea Conidi

**Affiliations:** 1 Department of Cell Biology, Erasmus University Medical Center, 3015 CN Rotterdam, The Netherlands; j.birkhoff@erasmusmc.nl (J.C.B.); d.huylebroeck@erasmusmc.nl (D.H.); 2 Department of Development and Regeneration, Unit Stem Cell and Developmental Biology, Biomedical Sciences Group, KU Leuven, 3000 Leuven, Belgium

**Keywords:** ZEB2, Mowat-Wilson Syndrome, neurodevelopment, intellectual disability, neural crest cells

## Abstract

After its publication in 1999 as a DNA-binding and SMAD-binding transcription factor (TF) that co-determines cell fate in amphibian embryos, ZEB2 was from 2003 studied by embryologists mainly by documenting the consequences of conditional, cell-type specific *Zeb2* knockout (cKO) in mice. In between, it was further identified as causal gene causing Mowat-Wilson Syndrome (MOWS) and novel regulator of epithelial–mesenchymal transition (EMT). ZEB2’s functions and action mechanisms in mouse embryos were first addressed in its main sites of expression, with focus on those that helped to explain neurodevelopmental and neural crest defects seen in MOWS patients. By doing so, ZEB2 was identified in the forebrain as the first TF that determined timing of neuro-/gliogenesis, and thereby also the extent of different layers of the cortex, in a cell non-autonomous fashion, i.e., by its cell-intrinsic control within neurons of neuron-to-progenitor paracrine signaling. Transcriptomics-based phenotyping of *Zeb2* mutant mouse cells have identified large sets of intact-ZEB2 dependent genes, and the cKO approaches also moved to post-natal brain development and diverse other systems in adult mice, including hematopoiesis and various cell types of the immune system. These new studies start to highlight the important adult roles of ZEB2 in cell–cell communication, including after challenge, e.g., in the infarcted heart and fibrotic liver. Such studies may further evolve towards those documenting the roles of ZEB2 in cell-based repair of injured tissue and organs, downstream of actions of diverse growth factors, which recapitulate developmental signaling principles in the injured sites. Evident questions are about ZEB2’s direct target genes, its various partners, and *ZEB2* as a candidate modifier gene, e.g., in other (neuro)developmental disorders, but also the accurate transcriptional and epigenetic regulation of its mRNA expression sites and levels. Other questions start to address ZEB2’s function as a niche-controlling regulatory TF of also other cell types, in part by its modulation of growth factor responses (e.g., TGFβ/BMP, Wnt, Notch). Furthermore, growing numbers of mapped missense as well as protein non-coding mutations in MOWS patients are becoming available and inspire the design of new animal model and pluripotent stem cell-based systems. This review attempts to summarize in detail, albeit without discussing ZEB2’s role in cancer, hematopoiesis, and its emerging roles in the immune system, how intense ZEB2 research has arrived at this exciting intersection.

## 1. Discovery of ZEB2

ZEB2, originally named SIP1 (and ZFHX1B), was discovered in a yeast 2-hybrid screening together with other candidate SMAD-interacting proteins (SIPs) from mid-gestation mouse embryos, using the transcription activation (i.e., MH2) domain of BMP-SMAD1 as bait [1]. At that time, the SMAD field had not yet identified low-affinity DNA-binding of receptor-activated SMADs, so the hypothesis was that their transcription regulatory function in the nucleus, where they accumulate together with SMAD4 in ligand-stimulated cells, occurred through co-operation and direct binding to other DNA-binding transcription factors (TFs). 

Further characterization of the partial cDNA from one of the positive prey plasmids showed sequence homology of SIP1 with the TF δEF1 (ZFHX1A, now ZEB1), previously discovered by the team of Hisato Kondoh in Osaka, Japan [2] and shortly after by many others. These TFs are the only two members of the small vertebrate family of zinc-finger E-box binding homeodomain (or ZEB) TFs. Related proteins with similar domain organization have been identified in the nematode *Caenorhabditis elegans* (Zag1) and the fly *Drosophila melanogaster* (Zfh1) [3,4,5,6,7]. It is not firmly shown that these proteins from invertebrates represent true orthologues of ZEBs, even when also considering their possible multiple action mechanisms resulting from their domain structure, such as for vertebrate ZEBs. For example, mouse ZEB2 is capable of restoring *Zfh1*-*null* cardiac phenotypes in the early fly embryo [5], but details are lacking about which domain/s of ZEB2 compared to those of wild-type Zfh1 itself is/are needed for this rescue. In addition, there is no evidence that Zeb2 is directly steering early cardiogenesis in vertebrates (including via phenotypic analysis of various *Zeb2* mouse mutants), except for its presence at E11.5 in epicardial cells [8].

## 2. ZEB2 and Mowat-Wilson Syndrome, and beyond

### 2.1. Mowat-Wilson Syndrome

Following an initial case report of a chr2q22-23 deletion that was difficult to delineate as a clinically recognizable syndrome [9], three teams [10,11,12] in independent studies of patients with severe intellectual disability, typical facial dimorphism and Hirschsprung disease (HSCR), identified *SIP1*/*ZEB2* haploinsufficiency as the cause of this new and rare, well-defined, monogenic syndrome, originally named severe mental retardation–Hirschsprung disease syndrome, and subsequently Mowat-Wilson Syndrome (MOWS; OMIM #235730; 1:50,000 to 70,000 live births) (https://mowat-wilson.org/ (accessed on 1 July 2021); [13,14,15,16]). 

**Figure 1:**
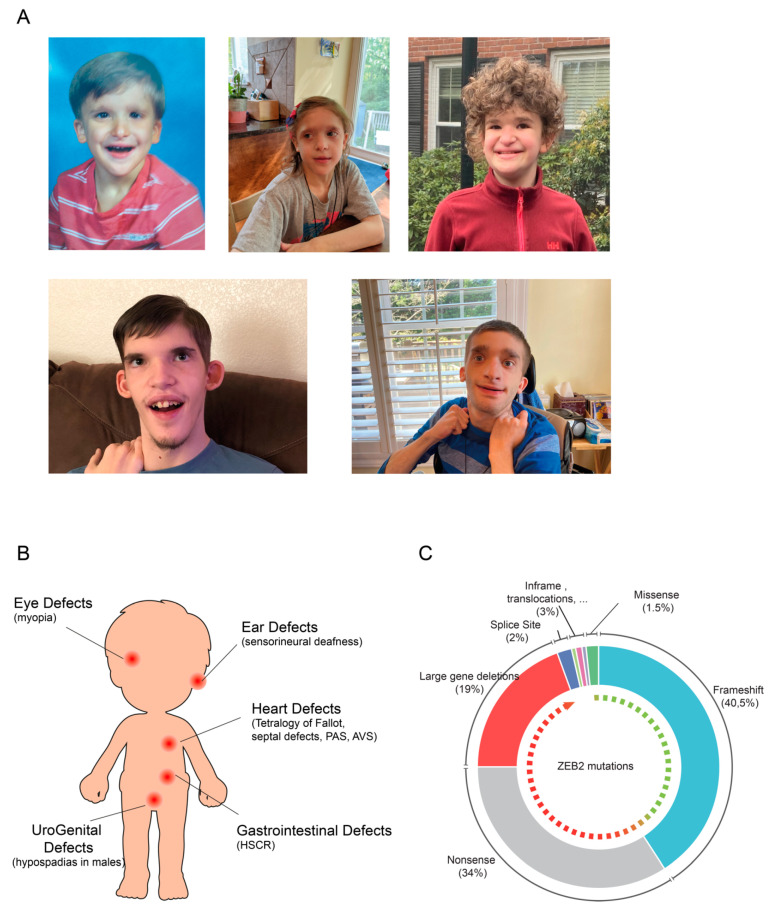
**Clinical features of MOWS patients and percentage of observed ZEB2 mutations:** (**A**) MOWS patients show typical facial features (courtesy of the Mowat-Wilson Syndrome Foundation); (**B**) congenital defects associated with MOWS; (**C**) Reported ZEB2 mutations. The green to red dashed arrow inside represents the severity of the disease with green being mild to red being very severe.

MOWS patients display a great variety of clinical features [17,18] with variable phenotypical penetrance [19] (Figure 1A). Patients present a delay in developmental milestones (sitting, walking, speech) and motor development. Various malformations in the central nervous system (CNS) often combine with developmental defects in the neural crest cell (NCC) lineage, which can all be observed by neuroimaging [20,21]. Almost 80% of the studied patients present anomalies of the corpus callosum and/or the hippocampus. Other anomalies are seen in the urogenital tract (hypospadias in males), kidney, eye (myopia) and heart (e.g., tetralogy of Fallot, septal defects, pulmonary arterial sling and aortic valve stenosis) or involve cleft palate, sensorineural deafness, as well as musculoskeletal and tooth anomalies. Al least half of the MOWS patients suffer from HSCR, which manifests by gastrointestinal defects as megacolon and constipation and/or obstruction of the bowel; about half of the patients also develop seizures and epilepsy [12,22,23,24] (Figure 1B). 

Up to date, mutant *ZEB2* protein-coding sequences have been determined for about 350 MOWS patients [21,23,25]. The majority and the severe cases of MOWS involve full gene deletions (in 19% of the patients), nonsense mutations (34%), or small insertions or deletions causing a frameshift leading to a pre-mature stop codon (40%). In the latter, nonsense-mediated messenger RNA decay (NMD) is likely the most significant factor, but if translated, this is expected to result in a nonfunctional, unstable protein [21,26]. Rare frame-shift mutations and in-frame deletions (5%) that affect only a short segment close to the C-terminus of the ZEB2 have also been identified, which in this specific case cannot be excluded to yield truncated protein [27]. Patients with such mutations generally show milder MOWS phenotypes, while the few missense mutations (only 1.5% of the total mapped cases thus far) cause the mildest phenotypes [23,28] (Figure 1C). 

### 2.2. ZEB2 Actions Studied Where Present

ZEB2 studies by many teams, initially in embryology and clinical genetics, and for both with focus on MOWS-relevant development of the CNS and peripheral nervous systems (PNS, including enteric nervous system, ENS), have meanwhile expanded towards many other fields. These are embryonic and adult stem cells, including differentiation into neurons, in vitro and in vivo [29,30,31], embryonic and adult hematopoiesis [32,33], and differentiation and terminal maturation of circulating or tissue-resident subtypes of immune cell in the adult [34,35,36,37,38,39]. ZEB2 is further studied in tumor initiation in concert with other regulators of epithelial–mesenchymal transition (EMT) [40] and metastasis [41,42], collagen fibrillogenesis in the dermal component of the skin [43], tight junction formation in cells and epithelial barrier function of the epidermis [44], and in the kidney as novel primary glomerular cystic disease gene [45]. In this review, we will focus on neurodevelopmental disorders and on important new lessons from other systems; we discuss ZEB2 neither in EMT/cancer nor in immune cells.

The research originally started with the documentation of *Zeb2* mRNA expression domains in amphibian embryos and mouse early post-implantation embryos, including as a component of transcriptional regulatory networks in embryonic neuroepithelium, and in the formation of the neural plate and subsequently brain cortex [46,47], presomitic mesoderm, and NCCs [48,49]. This was followed by further descriptive studies and forced overproduction (by RNA injection) of ZEB2 in amphibian embryos [50,51], including its promoting role in neural tissue formation [52]. Early post-gastrulation embryonic lethality of the general, homozygous *Zeb2*-knockout (KO) mouse was then reported [48,53], followed by *zeb2* knockdown (KD) experiments of the two genes in zebrafish showing early axial and neural patterning together with NCC defects [54], indicating the connection of ZEB2 with anti-BMP activity and ZEB2’s role in ENS, deficiencies that again link to MOWS. 

Conditional, cell-lineage or type specific, *Zeb2* KO (cKO) murine models were thus necessary (starting from *Zeb2^+^*^/*fl(*Δ*ex7)*^ mice; [53]). These cKO mouse models provide a main base for the content of this review, although meanwhile also cellular systems (embryonic stem cells (ESCs) and induced pluripotent stem cells (iPSCs)) are being developed. In more recent studies the cKOs were complemented with ESCs and mice enabling conditional (Cre-recombinase controlled) cell-type specific cDNA-based *Zeb2* overexpression (*Zeb2*-cOE) from the safe-harbor *Rosa26* locus [31,39,55]. The field also studied compound *Zeb1;Zeb2* mutant mice for few sites in the embryo where their presence overlaps, i.e., in somitogenesis [56,57,58], as well as compound *Zeb2^+^*^/*−*^ mutant mice with other mutant mice for Hirschsprung disease genes in order to study NCC-derived ENS cells [59,60]. Table 1 reports a list of ZEB2 mouse models associated with MOWS-like phenotypes.

These descriptive and functional studies have always been nicely complemented with biochemical studies, such as DNA-binding of ZEB fragments and subsequently full-length ZEB2, and ZEB2 binding to partners other than SMAD proteins. This longstanding dual approach firmly positioned ZEB2 as a multi-faceted TF [84,85]. For example, similar to ZEB1, ZEB2 transcriptionally represses many tested promoter-reporter combinations or endogenous genes in most assays, however both ZEB TFs can also directly activate (other sets of) genes. It is also clear that depending on cell type and context ZEB proteins display and combine domain and hence partner-dependent action mechanisms in the diverse cellular processes they (co-)control. 

Collectively, the homozygous *Zeb2*-cKO mouse models provided explanations for multiple defective cellular and developmental aspects of MOWS in humans. This was the case for CNS deficiencies, such as brain neuro/gliogenesis and guided migration of forebrain interneurons [47,64], as well as for eye lens malformation and for the resulting imbalance between cell types in the early and late retina [77,79,86], neurocristopathies resulting from aberrant craniofacial and ENS, as well as trunk NCCs [8,59,87], and reduced pain sensitivity [73,74]. 

In reverse, new studies, mainly and again using large panels of *Zeb2*-cKO mice together with transcriptomics of the *Zeb2*-defective cells, have enabled the identification of intact-*Zeb2* dependent genes, many of which are strong candidate direct targets for ZEB2. More recently, studies in immune (see above) or injury challenged mice documented novel roles for ZEB2 in vivo. Some of these new roles could prompt clinical geneticists to follow up MOWS patients longitudinally in new directions, for example with regard to remyelination by Schwann cells [67,68] and in epilepsy, caused by ZEB2 deficiency and its effects on guided migration in GABAergic interneurons [64] and their fate in the forebrain [63]. In addition to its cell-autonomous actions as a TF, cell non-autonomous effects have been documented for the first time in forebrain cortex pyramidal neurons [47], and thereafter in cardiac myocytes of the infarcted heart [81] and in endothelial cells of sinusoidal blood vessels in homeostasis, but also in anti-fibrotic action of ZEB2 in liver [82]. Such reports are expected to increase in number, positioning ZEB2 not only as a cell determination and maturation TF, but also as an important stem cell niche and tumor niche regulator. 

### 2.3. ZEB2 Levels Are Important, Too

Evidence is accumulating from various ZEB2 descriptive, perturbation and rescue studies that its mRNA and possibly protein levels need to be tightly controlled. Here, the better data are available for steady-state mRNA levels. For example, more detailed studies are needed to reveal the promoter-proximal and distant regulations that control the *ZEB2* locus in a dynamic chromatin context. In particular the mapping of *ZEB2* enhancers deserve more detailed studies, because *ZEB2* ranks in the very top of “super-enhancer” containing genes [88]. 

When carried out across species, such studies may also reveal subtle differences in *ZEB2* locus transcriptional regulation, in both spatial-temporal and strength aspects. This is nicely exemplified in the case of cancer: inappropriate *ZEB2* (over)expression correlates with bad prognosis of human early T-cell precursor leukemia, gastric cancer and melanoma, indicating that in healthy cells ZEB2 protein and activity need to be precisely controlled [41]. A spectacular and recent example, using human, gorilla, and chimpanzee cerebral organoids, revealed that ZEB2 promotes neuroepithelial transition, which also involves cell shape changes, and further that manipulation of ZEB2 and the resulting downstream effects can make human cells to acquire non-human (ape) organoid architecture, and vice versa. This demonstrated neuroepithelial cell shape as co-determinant for evolutionary brain expansion, with subtle differences in *ZEB2* expression between these species being an underlying principle [89].

Few studies have addressed ZEB2 protein (in)stability. Besides nuclear staining of ZEB2, also weak and diffuse cytoplasmic immunoreactivity has been observed in cell subpopulations in, e.g., in specific embryonic mouse forebrain locations [47]. Another intriguing possibility deserving in-depth studies, remains that *ZEB2* mRNA might be locally translated in neurons. Further, the E3-ubiquitin ligase FBXW7 directly binds and degrades ZEB2 in a phosphorylation-dependent manner, affecting ZEB2 levels in cancer tissues. Several teams have meanwhile started to characterize the ZEB2 proteome, which will improve our knowledge on the regulations of ZEB2 and partner recruitment, which both may be sensitive to post-translational modification. 

Based on insights in ZEB2’s action mechanisms, domain functions, co-operating proteins, direct target genes and/or the genes that are dependent on intact ZEB2 and regulatory elements in its locus, next-generation and elegant animal, cellular, and organoid models should now be designed to further study ZEB2. The same is true for MOWS patient inspired mutations, in particular the (still few) mapped missense or subtle mutations that affect the ZEB2 protein accordingly, mostly leading to milder phenotypes. In addition, for an expected growing number of mutations in mild cases of MOWS that do not affect ZEB2 protein, but do affect the timing and level of *ZEB2* transcription, cellular rather than animal models may be developed first. 

## 3. ZEB2 Gene and Protein Organization

**Figure 2:**
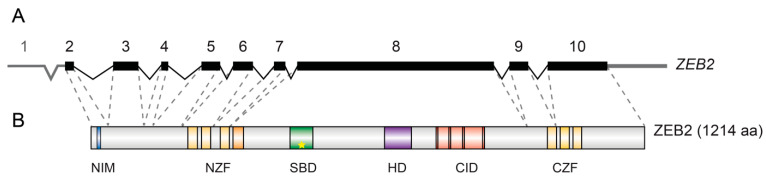
**ZEB2 Gene and protein architecture:** (**A**) human ZEB2 gene exon/intron is represented. Exon 8 encodes for about 60% of the protein. (**B**) ZEB2 protein structure. NIM: NuRD-Interacting Motif, NZF; CZF: N- and C-terminal Zinc Finger clusters; SBD: Smad-Binding Domain. The star in the SBD represents the essential residues in the SBD [91]; HD: Homeodomain-like Domain, CID: CtBP-Interacting Domain.

*ZEB2* maps to human chromosome 2q22 and encodes a ~140 kDa protein of 1214 amino acids (aa) through 9 exons (mouse *Zeb2* maps also to Cchr2; 1215 aa-long protein) (Figure 2A). Upstream of the first translated exon, the mouse *Zeb2* locus contains 9 untranslated exons (U1 to U9), which via alternative splicing are joined to the protein-encoding parts of the *Zeb2* transcript, without resulting in ZEB2 variants [90].

It was rapidly acknowledged that ZEB proteins are multi-domain proteins and elicit complex transcriptional controls in vertebrate development and in disease [92]. The very first indication that ZEB may serve one or more functions in a domain-dependent way came from early work by the team of H. Kondoh, using two different conventional *Zeb1*-KO mice wherein different parts of the *Zeb1* sequence were perturbed. Their homozygous *Zeb1-null(LacZ)* mice exhibit severe T-cell deficiency and skeletal defects of various lineages, whereas a *Zeb1* mutant allele obtained earlier, and that potentially could still produce a C-terminally truncated ZEB1 (but without CZF), showed only distorted composition of CD4 or CD8 expressing T-cells within the thymus and in peripheral lymph nodes [93,94].

ZEB proteins have a NuRD (nucleosome remodeling and histone deacetylation complex)-interacting motif (NIM); a homeodomain-like domain (HD) that deviates from a cognate DNA-binding HD and no longer binds to DNA; a C-terminal binding protein (CtBP)-interacting domain (CID) composed of 4 consensus interaction sequences for CtBP1/2 co-repressors; and two separated clusters of zinc fingers. In both the N-terminal zinc-finger (NZF, with 4 zinc fingers) and C-terminal zinc-finger cluster (CZF, with 3 zinc fingers), two zinc fingers are at least in vitro crucial for high-affinity DNA-binding [95]. Direct binding of any activated phospho-SMAD depends on a 51 aa-long segment (later shortened to 14 aa) of ZEB2, whereas ZEB1 does not contain this sequence [1,49]. In the SMAD-binding domain (SBD) of ZEB2, a tandem repeat of 4 aa (QXVX)_2_ is crucial for binding to all activated SMADS (star in Figure 2B; [91]), whereas full-length ZEB1 is considered not to be a direct SMAD binder [49,91]. This eliminates the intriguing possibility to construct ZEB2 variants that would lose binding to one SMAD class (e.g., the BMP-SMADs) whilst maintaining binding to SMADS of the other class (TGFβ/Activin-Nodal SMADs), or vice versa [1,91]. 

The difference between ZEB2 and ZEB1 regarding SMAD-binding remains intriguing, and is quite difficult to address experimentally. Time-consuming gene replacement studies or protein domain swapping in the mouse within the respective *ZEB1*/*2* loci, could be considered, but will ideally have to add cell-type specific functional analysis, seen the different and multiple phenotypes caused by either *Zeb2* or *Zeb1* deficiency as such. Otherwise, a single *Zeb* allele mutation in compound (non-*Zeb*) mutant mice could be considered [48,96]. Another aspect to consider is that *ZEB2* expression often precedes that of *ZEB1* in a given cell lineage or in tumors, or that their expression is complementary, e.g., in the developing mouse brain cortex where ZEB2 is present in the upper layers, while ZEB1 is in the lower layers [47]. 

Interpretation of the consequences of this duality in SMAD-binding may indirectly come from recent work in melanoma. First, a ZEB2-MITF-ZEB1 axis has been identified in melanogenesis and melanoma progression. Indeed, in addition to older evidence from NCC-specific *Zeb2*-KO mouse embryos where reduced MITF was observed [8], additional studies have confirmed that *Zeb2*-KO in this lineage results in downregulation of MITF. At the same time, this also brought new observations of downregulation of multiple melanocyte differentiation markers, accompanied by upregulation of *Zeb1* [76]. Second, ZEB2 in the melanoma models used here, is required for primary melanoma outgrowth and metastasis at secondary sites, phases during which upregulated/high ZEB2 levels are needed, whereas the gain of intermediate invasiveness depends on upregulated/high levels of ZEB1 and levels of ZEB2 should then be very low [42]. These intriguing observations for melanoma have been explained in various ways [83]. One extra explanation would be that this alternative use of ZEB1 versus ZEB2 is needed in the cells to escape from the SMAD-binding via *Zeb1* upregulation, and likely resulting in anti-SMAD activity of ZEB2. Inversely, high levels of ZEB2 may impact on negative regulation on subsets of shared SMAD/ZEB2 target genes in or around tumors where upstream ligands such as TGFβ and/or BMP co-create the tumor contexts. In this respect, but again experimentally difficult to achieve, it would be very valuable to re-establish similar mouse models wherein an SBD-inactivated version of ZEB2 (that leaves the rest of the protein intact) is encoded by (one or both of) its endogenous alleles. Furthermore, it is tantalizing to speculate whether one or more *ZEB2* mutant alleles encoding a subtle SBD in-frame/missense mutations would at all exist in disease, including congenital disease and, if so, whether this would present as MOWS or not. 

The last zinc finger of NZF, the SBD, HD and CID are all encoded by the largest *ZEB2* exon (exon8 in human; exon7 in mouse). This is the essential exon used for genetic inactivation in mice [53] and encodes for about 60% of the protein. The two zinc finger clusters are highly conserved between ZEB2 and ZEB1 (88% and 93% for NZF and CZF, respectively; [97,98]). ZEB proteins, initially tested as protein fragments and only later as full-length protein, bind DNA via NZF and CZF to two separated E2-like boxes, CACCT(G) and sometimes CACANNT(G) [1,2,95,99]. Although the integrity of both zinc finger clusters of ZEB2 is crucial for correct DNA-binding in vitro, the orientation and spacing in the DNA of the half-sites (and hence the target genes of ZEB2) can vary [95]. This is possibly due to the long linker region between the two zinc finger clusters in ZEBs, suggesting at the same time their likely flexible ZEB structure. 

ZEB2 interacts with various co-effector proteins. Already from the earliest studies, ZEB has been proposed as competitor for E-box-binding transcriptional activators simply by target-site occupancy, soon followed by the first reports of being a transcriptional repressor in most assays [99]. For this activity, co-repressor binding is considered important, e.g., with the NuRD complex [100]. In two patients with mild MOWS caused by mutations affecting the NIM, one being a subtle R22G missense variant, the only consequence is that ZEB2 interaction with the NuRD complex is lost [68,100]. Direct interaction of ZEB2 with CtBP at the repeated PLDLS sites within the CID has been claimed to render ZEB2 a more efficient transcriptional repressor [52,101,102,103,104]. CtBPs are not able to bind DNA in a gene/promoter specific context per se and thus require recruitment by DNA-binding TFs such as ZEB2 [105]. Importantly, CtBPs can retrieve HDACs (histone deacetylases) and HMTs (histone methyl transferases) towards repressed target gene promoters [106,107,108]. 

ZEB2 is covalently modified by Pc2-mediated sumoylation at two conserved sites, K391 and K866. which has been proposed to disrupt CtBP recruitment. This renders ZEB2 a less efficient repressor of, for example, *Cdh1* in EMT assays, while a sumoylation-*null* mutant for these two K residues is a very potent repressor for *Cdh1* [109]. However, when using full-length ZEB1 and ZEB2, it was found that interaction with CtBP is dispensable to repress transcription from the *Cdh1* promoter [110]. This is not the case for other genes modulated by TGFβ in the EMT assays, but these may not be direct target genes for ZEB2 [111]. 

Both ZEB proteins can also transcriptionally activate genes by co-operating with P300 coactivator and PCAF (P300/CBP-associated factor, also known as lysine-acetyltransferase 2B, KAT2B) through the associated HAT (histone acetyltransferase) activity. Interestingly, in this same study ZEB2-PCAF interaction was shown mutually exclusive with CtBP binding [51]. ZEB2 has also been reported as a transcriptional repressor for the TGFβ and NF-κB signaling pathways through disrupting the recruitment of P300 [111,112]. In addition, enhanced SUMO modification of ZEB2 by SUMO E3-ligase PIAS proteins at three other K residues (K5, K30, and in particular K108) weakens its inhibitory effect on TGFβ signaling. It was proposed that this SUMO-conjugated form of ZEB2 is impaired in its ability to disrupt ZEB2-SMAD complex interaction, and thereby also would indirectly influence the previously documented interaction between P300 and SMADS. This would leave ZEB2 behind as a non-SMAD bound strong repressor of TGFβ-Smad target genes such as *PAI-1* and *MMP2*, proposed thereby to strengthen TGFβ-regulated cell migration and invasion in the studied cell system [111]. 

However, this may not necessarily be the single action mechanism regulating ZEB2-Smad interaction at target genes. For example, activated SMAD1/5 and ZEB2 can be found together on their neighboring binding sites in the BMP-induced *Id1* promoter, which leads to strong downregulation of such *Id1* [86], hence reflecting the generation of anti-BMP(Smad) activity. This mechanism also explained how ZEB2 can overcome the BMP-mediated inhibition of embryonic CNS myelinogenesis, by directly promoting myelination from oligodendrocyte precursor cells downstream of the crucial OLIG1/2 TFs: here BMP-SMAD induced genes, which activate genes that inhibit myelinogenesis, are then repressed via ZEB2. Interestingly, here ZEB2 additionally and directly activates the transcription of *Smad7*, providing an extra negative feedback on BMP-SMAD signaling [62].

The current knowledge of ZEB2 domains potentially justifies starting designing CRISPR-Cas mediated subtle NIM and/or SBD mutations, even in vivo, and—depending on the cell type or state—document for which functions the intact respective domains are critical, including at the level of intact-domain dependent target genes. In addition, one could inquire if these domains in ZEB2 act (in)dependently from each other (additive, synergistically or antagonistically). In conventional mouse models, this would be easiest to address by checking whether embryonic lethality is identical in timing (i.e., E9.5; [48,53]) and nature (see above) as the conventional *Zeb2*-KO. However, adding a conditional, cell-type specific and ZEB2 domain-specific approach here may very well be needed, but is much more difficult. This also illustrates the current need to develop “easier” cellular models for this purpose and for the more subtle MOWS patient inspired mutations (missense mutations, enhancer mutations; see below) as well.

## 4. *ZEB2* Regulation

*ZEB2* gene transcription is controlled in various ways. First, the mouse *Zeb2* gene contains three alternative promoters, of which P2, located ~2.7 kb upstream of the start codon, is the most active one when tested in transfected cells [90]. Various *ZEB2* transcripts are expressed in different tissues in both mice and humans, and arise from cell type and tissue-specific alternative splicing. Indeed, the 5’UTR-encompassing gene region has multiple splice sites, which in part also originate from the various transcription initiation sites [113]. In addition, the *ZEB2* locus also encodes a natural antisense transcript that co-determines ZEB2 steady-state mRNA levels [114]. The *ZEB2* promoter-proximal region binds various TFs (e.g., Smad2, ETS1, HIF1α, POU/Oct and NF-ΚB, E2F1, FoxQ1 and FoxA2, Fra-1/AP-1; [115,116,117,118,119,120]. 

*ZEB2* is controlled in a tissue and time-specific manner by several (distal) enhancers (Table 2). In a unique transgenic rat model, an enhancer was mapped 1.2 Mb upstream of the *ZEB2* transcription start site (TSS), and found to be relevant to postnatal kidney development [121]. In mice, two other enhancers located 1.4 Mb upstream of the *ZEB2* TSS have been identified in the developing ventral forebrain. These enhancers are specifically active in cortical interneurons, and are suggested to be regulated by DLX1/2 [63]. In zebrafish, a total of 8 intron and downstream located *zeb2* enhancers located in introns and downstream of *zeb2* are active in the brain or specific brain areas, including in mid-/hindbrain, as well as in trigeminal ganglia and notochord [122]. Similarly, targeted chromatin conformation capture in neural differentiation of human ESCs mapped a cluster of active enhancers ~500 kb upstream of *ZEB2*’s TSS in a >3 Mb-long flanking gene desert, which is also found to be active in mouse ESCs submitted to neural differentiation [123] (and Birkhoff et al., unpublished results). These enhancers were thus most active in early NPCs derived from the PSCs, concomitant with increasing *ZEB2* steady-state mRNA expression [123], and decreased their activity in NPCs thereafter. 

Several micro-RNAs (miRNAs) control *ZEB2* levels in cultured cells, but also operate in vivo, e.g., in an adult neurogenesis compartment, the subventricular zone (V-SVZ) [124,125,126,127,128]. miRNAs from the miR-200 cluster can downregulate ZEB2 in epithelial cells and, together with miR-205, operate in and target ZEB2 in epithelial to mesenchymal transition (EMT) (such as for ZEB1; [129,130]), accompanying typical ZEB-mediated *Cdh1* downregulation [40,48]. In addition, the *ZEB2* 3’-UTR contains multiple targets for tissue-specific micro-RNAs, such as for miR-9, which regulates ZEB2 levels in postnatal rat brain cortex [131]. High miR-9 levels correlate with low ZEB2, and the increase in ZEB2 between postnatal day (P)2 and P5 goes together with immediate loss of miR-9 expression. 

Many more examples of miR-based ZEB2 control, and vice versa, have been reported in the literature. In mice, ZEB2 is a target of miR-192, which is important in the kidney, where increased expression of TGFβ leads to increased miR-192, which in turn results in decreased ZEB2 [132]. MiR-155 regulates ZEB2 in migrating and invasive colorectal cancer [133], and a miR-145-ZEB2-P53 axis operates in senescence of activated hepatic stellate cells [134], while a miR-145-ZEB2 axis acts in prostate cancer [135]. More ZEB2-controlling micro-RNAs are listed in Table 3, which omits also the role of the aforementioned ZEB2-AS RNA. This is not a complete list while new miR-ZEB2 associations are constantly being reported.

## 5. ZEB2 in the Development of Nervous Systems in Vertebrates

### 5.1. Initial Studies in Xenopus Embryos

The first studies of ZEB2 in embryogenesis used *Xenopus* embryos, in particular for studying mesoderm induction and neuroectoderm formation. Indeed, injection at the 2-cell stage of in vitro made mouse *Zeb2* sense RNA, and hence vast overproduction of ZEB in the early embryo, causes transcriptional repression of the panmesodermal *XBra* encoding the prototype member of the T-box family of TFs in all daughter cells of the injected blastomer(s) [1]. The *XBra* promoter contains an Activin/Nodal-responsive promoter segment in mesoderm induction, with a cognate ZEB2 binding site able to bind ZEB2 in vitro [95,160]. In a broader study of candidate regulatory elements in the *XBra* promoter, and using transgenic frog embryos to test spatial and temporal regulation of this promoter and the consequences of mutations in these control elements, the ZEB2 binding site proved critical to confine expression to the marginal zone of the embryo early in gastrulation. This became apparent from the expanded, disturbed expression domain of *XBra* upon ZEB2 binding site mutation, but this deviation of the *XBra* expression then rapidly returns to the normal domain. This demonstrates that the putative ZEB2 binding site in vivo (and that binds ZEB2 in vitro; [95]) only serves *XBra* regulation in a short time window. Furthermore, this is compatible with the observed brief overlap of *XBra* and *XZeb2* mRNA domains, but these rapidly segregate with *XBra* being expressed in prospective mesoderm and with *XZeb2* from stage 10.5 in anterior neuroectoderm (in particular in its sensorineural layer), neural plate, NCCs at the border of the neural plate, and migrating NCCs, including these leaving from the dorsal side of the closing neural tube [49,50]. 

The ZEB2-mediated repression of *XBra* is highly specific and was studied further by fusing the entire *XZeb2* open reading frame to the strong transcription activation domain of the viral TF VP16 (*X*Zeb2-VP16, [50]). In this study from Papin and co-workers, embryos that overproduce *X*Zeb2 from injected RNA failed to complete gastrulation, and this results in greatly reduced posterior structures by the tadpole stage; in Activin-treated animal caps such overproduction of ZEB2 inhibited Activin-induced convergent extension as essential cellular process in gastrulation [50]. These results were in line with those of other teams that used dominant-interfering *X*Bra-EN^R^ (repressor domain of Engrailed) fusions, which turn *X*Bra in a transcriptional repressor of its cognate target genes [161], altogether suggesting that *X*Zeb2 indeed exerts direct inhibitory activity on *XBra* transcription. 

In subsequent experiments, Papin and co-workers (2002), after demonstrating that overproduction of *X*Zeb2-VP16 is now a strong activator, but needed intact NZF and CZF for DNA-binding, showed that RNA-injection resulted in maintenance of endodermal genes and *XBra*. However, neither activation of other mesodermal markers (such as Spemann Organizer *XGsc*) is seen at the early gastrula stage nor prospective muscle-specific genes at late neurula stages as well as the normal, increased levels of neural markers such as *NCAM*. Such embryos fail to complete gastrulation and the anterior structures were absent by tadpole stages. These defects can be rescued by co-injection of wild-type *XZeb2* sense RNA. In a next series of experiments in *Xenopus*, it was shown that during the establishment of the neuroectoderm, ZEB2 inhibits BMP-SMAD signaling by directly repressing *BMP4* transcription via a ZEB2-CtBP-complex. In normal embryogenesis this results in downregulation of epidermal markers and consequently induction of neural genes such as *Sox2*, *Sox3* and *NCAM*, leading to further neuralization [52,162]. 

Taken together, these results strongly suggest that *X*Zeb2 is required for formation of anterior neuroectoderm. This key observation, together with *Zeb2* expression in NCCs, prompted many to study ZEB2’s role in CNS and PNS development. 

### 5.2. Modeling Neurodevelopmental Aspects of MOWS in Mice

#### 5.2.1. General *Zeb2*-KO

The functions of ZEB2 in cell fate decisions at important stages in amphibian embryogenesis, and the strong possibility that ZEB2’s actions downstream of and/or modulating, e.g., embryonic Nodal-Activin/BMP signaling, would require conditional loss-of-function approaches in the mouse. The establishment through intense collaboration of cKO founder mouse was a next critical step in the ZEB2 field. This was possible through “floxing” (*fl*) by homologous recombination the large, critical exon7 of *Zeb2* (*Zeb ^fl(ex7)^*) in mouse ESCs [53], which can be deleted by Cre (creating the *Zeb2*^Δ*ex7*^ KO allele). 

*Zeb2^+^*^/*fl(ex7)*^ mice were crossed with *(Ad)EIIa*-Cre mice, which carry a Cre-transgene under the control of the Adenovirus EIIa promoter, among other tissues active in the germ cells, to obtain homozygous *Zeb2*-KOs. Such embryos have visible defects from E8.5 onwards, correlating directly with the primary *Zeb2* expression sites, with a neural tube that fails to close, no longer having a sharp boundary between neural plate and the rest of the ectoderm, and neuroepithelium that fails to downregulate *Cdh1*. Furthermore, these mice miss the *Twist+* first branchial arch. The *Sox10+* (NCC identity) and *AP-2+* and *Hfh2+* migrating cranial NCCs do form, but fail to migrate, and *Sox10+*, and subsequently *Msx1+* vagal NCCs were absent. The embryos are also visibly smaller at E9.5, did not undergo turning, and die in utero around this stage. 

Heterozygous *Zeb2*-mutant mice did not visibly develop histomorphological phenotypes, also not at the E8.5-9.5 stage, but this likely depends on genetic background. Indeed, inducing the *Zeb2*^Δ*ex7*^ mutation in germ cells and deriving as such a pure C57BL/6 line enabled for the first time to maintain over longer time *Zeb2^+^*^/Δ*ex7*^ mice in this background [65]. Such adult mice exhibit craniofacial abnormalities, and defects in some brain regions (corpus callosum, parvalbumin neurons in brain cortex) and behavioral activity (reduced motor activity, increased anxiety and impaired sociability). Some of these defects are MOWS-like and are not seen in the closely related ICR and outbred backgrounds. 

#### 5.2.2. MOWS and Neurodevelopmental Relevant *Zeb2* Expression Domains in Mouse Embryos

In E8.5 mouse embryos, *Zeb2* mRNA is detected in the neuroepithelium, where its levels follow the spreading of the maturation of the neural plate and the delineation of its borders. *Zeb2* is, like in *Xenopus* and zebrafish embryos, highly expressed in premigratory and delaminating NCCs of the cranial region of the embryo, and subsequently in branchial arch mesenchyme and in postotic, vagal NCCs [48]. 

In addition to *Zeb2* mRNA and protein localization studies as part of most loss-of-function studies in cell types and embryonic locations of interest (see below), another mouse line with a EGFP knock-in in the *Zeb2* locus [163] has proven particularly useful as reporter for brain development in general as well as recent studies of ZEB2 in endothelial cells that line hepatic capillaries in the liver sinus [82]. Because we focus here on Zeb2 functional analysis of CNS/PNS development in the mouse and also include adult neurogenesis, and because of the connection to certain aspects of MOWS, we recall here EGFP-based confirmation of ZEB2 presence in pyramidal neurons of the hippocampal dentate gyrus, and in neurons in the brain cortex, where in layer 5 *Zeb2*-driven EGFP was complementary to CTIP2+ neurons, possibly in corticospinal neurons. In addition, the reporter could be detected in oligodendrocytes (of the corpus callosum and fimbria), in Bergmann glial cells of the cerebellum, in the olfactory bulb, and in serotonergic and dopaminergic neurons of the raphe nuclei in the brainstem [163].

#### 5.2.3. *Zeb2*-cKO in the Neural Crest Cell Lineage

NCCs are a transient embryonic cell lineage originating from the border between the ectoderm and the neuroectoderm under the control of the right dose of BMP-SMAD signaling, all along the anterior-posterior axis of the embryo at its dorsal midline [164,165,166,167,168]. These cells undergo EMT, delaminate, migrate extensively, and differentiate into ectomesenchymal cell types, including neurons and glial cells of the PNS, pigment cells, smooth muscle cells, craniofacial cartilage and bone [169,170], and dental pulp cells [171]. By directly inducing EMT via the repression of *Cdh1*, and by being an anti-BMP factor, ZEB2 may thus contribute to the formation and regional features of NCCs.

At E8.5 in mouse embryos, *Zeb2* mRNA/protein is also present in premigratory and migrating NCCs, and those populating the mesenchyme of the branchial arches. At E9.5, the first branchial arch (which becomes the maxilla), the palate, and the mandibula are *Zeb2*-negative, but Schwann Cell precursors associated with the nerves that extend from the trigeminal (V) ganglia into the first branchial arch are *Zeb2*-positive (*Zeb2*+). At E10.5, *Zeb2* mRNA is found in the trigeminal (V) and the facio-acoustic (VII-VIII) ganglia, the ganglia of the glossopharyngeal (IX) and vagal (X) nerves, and dorsal root ganglia (DRG) in the trunk region [8]. Interestingly, at E11.5, *Zeb2* re-appears in the NCC-derived mesenchyme of both the maxillar and mandibular process. At E12.5 a local upregulation of *Zeb2* can be spotted in the dental mesenchyme of the molar tooth primordia. At E15.5 the connective tissue of the papilla in the in the follicles of the vibrissae as well as the surrounding mesenchyme is *Zeb2*+. These accurate *Zeb2* expression domains prompted investigation of ZEB2 function in NCCs in various ways.

#### 5.2.4. Neurocristopathies Reminiscent of MOWS

The *Wnt1*-Cre approach, which allows to inactivate *Zeb2* in NCC precursors from 4-somite stage onwards, resulted in embryonic lethality with incomplete penetrance from E11.5 onwards as well as death shortly after birth. The craniofacial and gastrointestinal malformations that were documented clearly explained these aspects with MOWS patients. For example, at E14.5 these *Zeb2*-cKO embryos showed distinct facial abnormalities, i.e., reduced muzzle mesenchyme with lack of whisker follicles, shorter snout, and wide-open eyes. In the *Zeb2*-cKO newborns, the nasal and premaxilla bines are hypoplastic, and ossification is incomplete and appears fragmented. The molar socket is absent, whereas incisors are normal. In the skull, the mandibles are not curved, and the squamous parts of the frontal and parietal bones lack ossification towards the metopic region. This results in broadened and sagittal and metopic sutures, but not of the coronal sutures. Additionally, the ear region of the skull appears affected, as the retrotympanic process of the squamous bone is severely reduced in size, while in the inner ear the tympanic ring and gonium are truncated. 

In addition to these clinically recognized craniofacial (and gastrointestinal, see below) defects, other developmental defects were found. These were anemic yolk sac despite the presence of blood vessels, pooling of blood in peripheral vessels, and heart and liver, and signs of acute heart failure from E11.5–12.0, as seen from the hemorrhage on the ventricles, which could indicate rupture of myocardial tissue, but also absence of epicardial cells in otherwise, at E11.5, no detectable dysfunction of ventricular or compact myocardial. An early arrest of melanoblast development at E11.5 onwards is also seen, for example in the head and rostral trunk region. Additionally, reminiscent of MOWS, developmental defects in the adrenosympathetic lineage, and sympathetic and parasympathetic anlagen were found [8]. Many of these observation in this NCC-specific *Zeb2*-cKO mouse embryos and newborns thus fit with malformations in MOWS patients, including pigmentation defects in few of them, present cupped ears with upturned lobules, wide nasal bridge, deep-set eyes, hypertelorism, pointed chin, prominent rounded nasal tip, and a high incidence of microcephaly, malpositioning of teeth and/or delayed tooth eruption [172].

#### 5.2.5. Dorsal Root Ganglia and Pain Sensing

Importantly, ZEB2 is also found at high levels during the condensation of NCCs in DRG at E9.5 and, from E10.5 on, high levels of nuclear staining are maintained within the differentiating DRG as well as in cells aligning peripheral nerves, reflecting expression in axon-associated Schwann cell precursors. Van de Putte and co-workers (in the context of [8]) had also observed in NCC-specific *Zeb2*-cKO that a late-migrating wave of NCCs bound for the DRG did not form. However, glial cells of the PNS are also generated by NCCs. These comprise satellite cells, which are the glial cells associated with neuronal cell bodies in the ganglia, and Schwann cells, which mature in myelin and non-myelin-forming cells that wrap the peripheral nerves. Therefore, they also addressed the development of boundary cap cells, the major source of the late supply of *TrkA+* nociceptive and thermoceptive neurons that contributes also to a subset of satellite glial cells [173,174]. Between E10.5–15.5, these actively proliferating cells are the only trunk cells that do express *Egr2* (*Krox20*), and these cells could not be found between E10.5–12.5, where then they would be associated with the vagal nerve (E10.5) and also in cranial and trunk motor exit points and dorsal root entry zones (E10.5–12.5). These results prompted them to subsequently address ZEB2-dependent DRG function, in particular with regard to nociceptive neuron excitability and pain sensitivity using *Zeb2^+^*^/Δ*ex7*^ mice, and ZEB2 function in Schwann Cell precursors and adult (re)myelination (see below).

Phenotypic characterization of adult *Zeb2^+^*^/Δ*ex7*^ mice identified reduced thermal pain responses whereas mechanical pain was unaffected [73]. Electrophysiological measurements revealed as underlying mechanism a reduced spike gain only in capsaicin/heat-sensitive DRG neurons, which was accompanied by an up-regulation of persistent Na^+^-channels and a decrease in delayed rectifier K^+^-channels. Further modeling of the electrophysiological results also suggested consequences for signal propagation from the periphery to the spinal cord, and it was proposed that ZEB2 regulates thermal pain sensitivity by controlling the transduction properties of nociceptive primary sensory neurons in a novel manner, namely via changes in DRG voltage-gated ion channels. In a follow-up study [74], where the role of ZEB2 in inflammatory and neuropathic pain was investigated in various challenged adult *Zeb2^+^*^/Δ*ex7*^ mice, the hypoalgesic phenotype of these mice was proposed to originate from the inflammatory component (but not from neuropathic pain) and due to ZEB2-dependent development of primary sensory DRG neurons, in particular the C- and Aδ fibres. This also suggests that the under-reaction to pain, sometimes (but not systematically, as this is difficult to address in the clinic) observed in MOWS patients, results from a reduced responsivity to nociceptive stimulation rather than an inability to communicate discomfort.

#### 5.2.6. The ENS and HSCR

Congenital megacolon/HSCR occurs in half to two-thirds of MOWS patients. NCCs bound for the gastro-enteric nervous system (ENS) originate from the postotic hindbrain adjacent to somites 1–7 (vagal NC) and possibly the sacral NCCs that emerge caudal to somite 24. The former innervates the esophagus, stomach, and small and large intestine, and the latter is proposed to colonize the hindgut. At E10.5 and later, ZEB2 protein is present in single cells within the mesenchyme of the fore-/mid-gut wall. From E12.5, high ZEB2 immunoreactivity is present in the submucosal and myenteric plexus of the stomach, and along the entire intestinal tract. Severe intestinal agangliosis is seen in NCC-specific *Zeb2*-cKO mouse embryos at E18.5. The defect involves the distal colon (reflecting sacral NC defects) and extended past the ileocecal junction into the small intestine, whereas the stomach and the rostral part of the duodenum were innervated. Further analyses revealed that NCC derivatives entered the foregut, but stall their migration already around E10.5, causing failure with the enteric precursors to massively populate the gastro-intestinal tract beyond the distal half of the stomach [8]. 

The aforementioned research on ZEB2 in NCC-derived ENS, together with novel insights in causal genes of HSCR around that time (for review, see [175]), and similarities in defects in other syndromes that originate in NCC-derived ENS (e.g., *SOX10* in Waardenburg-Hirschsprung disease) inspired for phenotypic analysis of double *Sox10;Zeb2* mouse mutants. Briefly, such mice have more severe defects in both maintenance and differentiation of progenitors in the developing ENS [59]. Similar controls are exerted on the progenitors by EDN3 (endothelin-3), which acts via its receptor EDNR (type B). Consequently, a similar approach was used using again double mutant mice (*Ednr^ls^* mice, a spontaneous recessive mutant incapable of converting EDN3 to its active form; and *Zeb2^+^*^/Δ*ex7*^ mice), with EDN3 preventing wild-type ZEB2 cells to undergo neuronal differentiation. Here, also cultures of enteric progenitor cells from these mice were used, and rescue experiments in these using the respective cDNA-based transgenes. In addition to the more severe enteric anomalies in the double mutants, other valuable conclusions were that two intact alleles of *Zeb2* are required for EDN3 to inhibit neuronal differentiation, because one such copy (*Zeb2^+^*^/Δ*ex7*^) only enables partial EDN3-mediated differentiation. The underlying reason is that SOX10 and ZEB2 directly activate the *EDNRB* promoter.

#### 5.2.7. *Zeb2*-cKO in the Developing Forebrain 

*Zeb2*-cKO mice for brain development studies were based on mapped *Zeb2* mRNA/ ZEB2 protein locations and clearly benefited from the availability of well-characterized, suitable Cre-driver lines. Shortly after the onset of corticogenesis at E12.5, ZEB2 is present in the ventral part of the forebrain in the ventricular zone (VZ) and dorsally in postmitotic cells of the cortex. ZEB2 staining increases at both locations at E15.5, with clear staining in the intermediate zone and cortical plate of cortex and hippocampus at E16.5 and E18.5 [47,61]. 

Specific KO of *Zeb2* in cortical precursors (using *Emx1*-iresCre) results in lack of the entire hippocampal formation, as the result of a combination of significant death of differentiating cells and decreased cell proliferation in the prospective hippocampus region and its dentate gyrus (DG) [61]. These mutant mice are viable, often reach the juvenile stage (3–4 weeks of age), are generally growth retarded, have smaller brains, and hippocampus and corpus callosum are missing. Further analysis time the first visible deficiencies in the (smaller) hippocampus from E15.5, with reduced CA1 and CA3 fields and an almost absent DG. At that time, micro-array based detection of changed genes was used for the KO mice, which indicated changes in the mRNA levels of the Wnt antagonist *Sfrp1* (in particular its high and ectopic expression in the KO mice) and *Jnk*, also known as a non-canonical Wnt effector (which was downregulated in the KO mice). These observations were in line with previous reports on Wnt3a signaling, its components (e.g., *Lef1*/*Tcf*, *β-catenin*, *Frz9*), and some of its targets (*Emx2*), and thus playing a relevant role in hippocampus development. *Sfrp1* was suggested as a possible direct target gene of ZEB2 and to result in their mutual expression, however based on the expression of the mRNA of Wnt components, it was concluded that canonical Wnt signaling was not impaired in the mutant hippocampus, but activated forms of JNK1-3 levels (and not their total levels), which depend on main effectors such as CamII (Ca^2+^-dependent calmodulin kinase II), had dropped significantly. This would identify ZEB2 function here as a positive regulator of noncanonical Wnt signaling. ZEB2 may repress the general Wnt inhibitory *Sfrp1* gene directly. Therefore, upregulation of the latter in the KO mice leads to inhibition of JNK activity, and brings about the aforementioned proliferation and apoptosis defects.

Three different Cre-drivers were subsequently used to inactivate *Zeb2* in the entire embryonic CNS (*Nestin*-Cre), in its neocortical precursors (*Emx1*-iresCre; see above) and (mainly) in postmitotic neurons (Nex-Cre) [47]. *Zeb2* removal was found to shift the timing of cortex upper layer formation forward, leading to expansion of these layers at the expense of the deeper layers. ZEB2 actions also include the control of the timing of glial precursor specification from E16.5 onwards, which continues after birth causing more OLIG2+ cells, and at P2-P4 yielding increased numbers of differentiated, GFAP+ astrocytes. Neither differences in cell-cycle length nor accelerated mitotic exit of neurogenic progenitors are the cause, but direct effects on cell-fate choice were. RNA-sequencing (RNA-seq) indicated changed levels of *Ntf3* (Neurotrophin-3), which is likely a direct target gene of ZEB2, and *Fgf9* (Fibroblast growth factor-9), accompanying stronger staining of downstream MAPK-ERK type kinases, in particular phospho-MAPK p42/44 at E14.5 in the VZ, where ZEB2 is absent. Altogether, this indicates a cell non-autonomous action of ZEB2 in the upper layers of the cortex, i.e., a neuron-to-progenitor feedback signaling. ZEB2 does so by controlling *Ntf3* and Fgf9 levels feeding back to the VZ, with impact on timing on neurogenesis and a bit later gliogenesis. In the *Zeb2*-KO mice, premature onset of *Ntf3* expression already at E12.5 and of *Fgf9* at E16.5 thus precedes the detected cell-fate change from deep to—and in favor of—upper layer neurons at E12.5–E13.5 and coincides with a switch from neuron production of glial cell formation at E16.5–E17.5.

Both the aforementioned ZEB2 presence in the prospective basal ganglia of the forebrain (including the MGE, LGE and CGE), but also phenotypic analysis of the *Nestin*-Cre;*Zeb2*-cKO used by Seuntjens and co-workers (2009 [47]; see above), as well as seizures and epilepsy in MOWS patients, then prompted investigation of ZEB2 in interneuron generation in the ventral telencephalon and in migration of these cells [176]. Sorted E14.5 interneurons from a, *Nkx2.1*-Cre (active in MGE cells), were particularly useful to phenotype these *Zeb2*-deficient cells using RNA-seq for the first time. Taken together, these important studies revealed hampered tangential migration in *Zeb2-null* cortical interneurons in the telencephalon of which the early regionalization is not affected. In addition, the use of embryonic forebrain slices ex vivo, combined with electroporation-mediated and vector-based transgene expression (including rescue *Zeb2*-cDNA and reporter-cDNA), enabled to demonstrate that ZEB2’s action in this case is cell-autonomous. RNA-seq, in addition to documenting (un)changed levels of many TF mRNAs, revealed upregulated as well as downregulated genes in *Zeb2*-cKO MGE cells, irrespective as to whether they are direct targets for the repressor or activator activity of ZEB2, respectively. 

Most importantly, the results strongly indicated upregulated levels of genes annotated to “axon guidance”, in particular “signaling by transmembrane receptors”, which is in line with the cell-autonomous mode of action of ZEB2 in these cells. In the latter gene ontology (GO) term, the mRNA for Netrin receptor family components *Dcc* and *Unc5* were particularly upregulated in the KO cells. Both receptors can bind Netrin on their own, but Netrin can also bind to DCC-UNC5 heterodimers, which makes this Netrin-receptor system to transmit either a long-distance attractive cue (via DCC) or (with UNC5 action) a long or short-range repulsive cue. Increased UNC5 levels, when knocked down in electroporated slices of mutant forebrains, were able to rescue aberrant migration of the cells. Taken together, these results indicate that ZEB2 is needed for local tuning of guidance cues in interneurons from the MGE mantle zone in the developing brain, but it remains to be established which Netrin or possibly some of the novel FLRT ligands are the endogenous ligands here that cause misrouting of these mutant cells. Importantly, but not followed up by additional studies yet, other GO terms that are deregulated in these *Zeb2*-cKO MGE cells are genes involved in synaptogenesis and synaptic plasticity.

Meanwhile, McKinsey et al. (2013) [63] and van den Berghe et al. (2013) [64], using similar approaches, had shown that removal of ZEB2 prevents repression of *NKX2.1*, which has been suggested by McKinsey and co-workers to cause a change in interneuron cell fate from cortical to striatal types. Most importantly, the observed eventual deficit of GABAergic inhibition in these mouse models are supposed to underlie focal and absence seizures [177,178], in particular in MOWS patients [14]. In this respect, and despite preliminary data in these mouse models on neuron connectivity and projection, it remains to be established whether modification of cell identity or these circuitries, which depends on brain area, shifts the balance from anti- to pro-epileptic [179].

Other mouse models demonstrated the importance of ZEB2 for axonal growth and projections, as different axonal pathologies are described in MOWS patients [14,180]. In one such model *Zeb2*-cKO was obtained in pyramidal neurons, causing the complete lack of intercortical connections in the corpus callosum, the anterior commissure and the corticospinal tract [66]. Here, ZEB2 is needed for regulation of the production of the microtubule-binding protein Ninein, which is essential for axonal growth. Hence, ZEB2 is needed to control the timing and growth of axonal projections in the neocortex.

#### 5.2.8. ZEB2 in the Formation and Output of the Adult Neurogenic V-SVZ Compartment

Postnatal neurogenic zones, such as the V-SVZ (ventricular–subventricular zone) that lines the lateral ventricle walls, have an embryonic origin. Interestingly, a fraction of radial glial cells (RGCs) in the forebrain LGEs (lateral ganglionic eminences) and pallium becomes quiescent [181,182]. ZEB2 was studied in LGE-derived neurons for LGEs using mainly the *Gsh2*-Cre driver [64], hence ZEB2 co-steers the anlage of part of the V-SVZ. After birth, these cells are reactivated and differentiate into the different cell types of the V-SVZ, which in the mouse are architecturally grouped, and produce supportive cells (ependymal or E cells) or RGC-like neural stem cells (NSCs or B1 cells; [183]) of the V-SVZ. The latter cells generate C cells (transit amplifying cells), which give rise to A cells (immature, including migratory neuroblasts). As cells migrate via the rostral migratory stream (RMS) to the olfactory bulb (OB) and emanate as network-integrated different types of interneuron [184]. The V-SVZ and its RMS expresses miR-200 class of micro-RNAs (that target Zeb2; [124]) and, at the time B cells are set aside in the embryonic brain, ZEB2 mRNA/protein is present in the ventral telencephalon, including the aforementioned LGE [64]. In *Gsh2*-Cre;*Zeb2*-cKO LGEs, about 70% of postnatal V-SVZ cells are detectably targeted at E14.5, and this is confirmed in P2 and P5 mice. Pronounced defects are seen in these KO mice in OB development, for they have more scattered distribution of the various cell types and. Additionally, at P17-P18, the fewer OB interneurons results in OBs that are 70% smaller than normal. This suggested an important role of ZEB2 in postnatal neurogenesis.

Further analysis confirmed the hypothesis that the smaller OB would be due to reduced numbers of DCX+ A cells that arrive in the OB, and which look morphologically aberrant as well [30]. This is not due to misrouting of interneurons before they enter into the OB, but seems to originate from decreased production and changed survival of cells in the V-SVZ. Electroporation of a CAGGS driven-Cre vector (and a reporter vector) in brains of *Zeb2^fl(ex7)^*^/Δ*ex7*^ mice results in a drastic drop of *Zeb2*-KO neuron numbers in the OB. After comparative RNA-seq, this is likely due to strongly upregulated *Sox6* (a strong candidate as direct ZEB2 target) in *Zeb2-null* cells, and not to delayed migration (when looked at P2 as well as P56) as seen also upon embryonic KO (see above). Other experiments also traced ZEB2 action mainly in C cells and their progeny. Taken together, ZEB2 has a cell-intrinsic role in regulating the output from the early-postnatal V-SVZ. In addition, ZEB2 removal also affects the maturation of various OB interneuron cell types. 

Importantly, this study was also one of the first to show that ZEB2 proteins with domain mutations (in the NIM or in the SBD) bear different rescue capacities in both quantitative and qualitative terms in electroporated mutant *Gsh2*-Cre;*Zeb2*-cKO brains. This is possibly due by impacting on the precise balance between repressor activity of ZEB2 on a set of genes versus activator activity on another set of genes. Rescuing with a ZEB2-SBD mutant leads to increased numbers of OB interneurons, indicating that ZEB2-SMAD co-operation is crucial for tightly regulating the output capacity of niche progenitors. The ZEB2-SBD mutant may render the cells incompetent to regulate SMAD signaling, potentially leaving the cells longer in proliferation. Although these are important results also for brain injury repair, it remains to be tested whether ZEB2 is critical to adult neurogenesis in conditions of unilateral brain injury.

A connection may emerge with reactive gliosis, which is known to involve upregulated BMP actions in this process [185,186]. Interestingly, ZEB2 has been identified in the mouse as regulator of the astroglial response to transient ischemic stroke or spinal cord injury, likely through its EMT regulatory functions. Removal of *Zeb2* from mouse astrocytes attenuates reactive gliosis and delays recovery of motor function [70]. This ZEB2 effect is interesting and could be tested also in the PNS, e.g., the ENS, where possibly under co-control of BMPs also the generation of new enteric neurons from glial precursors has been proposed as novel avenue for repair, further supported by studies in injured mouse cerebral cortex [187]. 

Taken together, ZEB2-co-controlled astrocyte activation and reactive gliosis, including in a context of multicellular responses (including microglia; [188]) to nervous system damage and disease, is an exciting new ZEB2 research line to explore in mouse and zebrafish models for (repetitive) injuries and pathologies of the brain [189], spinal cord [190], retina [191] and ENS [192,193], and perhaps, more speculative, also in blood vessel disease caused by endothelial dysfunction, e.g., in the brain and in neuroretinal degeneration [194]. 

#### 5.2.9. ZEB2 in Midbrain, Hindbrain, and Spinal Cord

##### Midbrain

In contrast to the extensive ZEB2 studies in forebrain, its neurodevelopmental role in the rest of the CNS appears under studied. From a screen to identify and map the location of cell-intrinsic regulators of midbrain dopaminergic (mDA) neurons in the CNS, in particular of axon growth and target innervation, Hegarty and co-workers picked up ZEB2 as a candidate in the midbrain during the period of striatal innervation. In the mouse CNS, high numbers of mDA neurons are found in the brainstem in the substantia nigra that projects axons to the striatum after their generation between E10–E12. Their axons already extend rostrally from E11 and reach the striatum the next day, and by E16 innervate its rostral. The first 3 weeks after birth, cell death is part of pruning the excess mDA neurons that are formed [69,195]. 

*Zeb2* mRNA expression is dynamic in the mouse ventral midbrain (VM), but increases from E10 and peaks at E12, followed by a strong decrease till at least E18. Intriguingly, many components of the BMP system significantly increase between E12 and E18, and BMP-SMADs are active during mDA axonal growth. Further KD and OE experiments in (rat) ventral midbrain neurons in vitro and descriptive studies in vivo indicated that the increased ZEB2 amounts negatively regulate hosphor-Smad1/5 levels here, and that downregulation of *Zeb2* is necessary and sufficient for BMP-SMAD activated axon growth, but high *Zeb2* levels could not affect basal levels of neurite outgrowth in rat E14 VM neurons. Interestingly, KD of the ZEB2 partner CtBP1 in rat E14 VM had the same neurite-outgrowth stimulatory effect as KD of ZEB2, suggesting that ZEB2 and CtBP co-operate as transcriptional repressors for inhibiting axon growth. However, basal levels of ZEB2 are needed to repress activated BMP-SMADs below a growth-promoting threshold. To further investigate this in vivo, *Nestin*-Cre;*Zeb2*-cKO mice were used [47]. While their mDA neuron numbers at E12.5 and E16.5 do not differ from the controls, they do have higher fiber density and thus show dopaminergic hyperinnervation in the striatum at E16.6, when normal striatal innervation is ongoing. These mice did not show a difference in the amount of mDA neurons compared to control mice, but they did show hyperinnervation of mDA neurons in the striatum [69]. 

These results were confirmed by Yang and co-workers (2018) [195], using an ZEB2 OE approach of in utero electroporated E11.5 mouse brain, resulting in a decrease in migrating mDA neurons; in addition, ZEB2 was confirmed to act as negative regulator of BMPR-SMAD1/5 activity, but is also a target of miR-200c [195], which has been proposed to downregulate and control ZEB2 levels during mDA differentiation, in particular preventing premature mDA differentiation, migration, and innervation into the striatum. This is again an example of needed precise control of *ZEB2* mRNA levels. Clinical geneticists have proposed that this work may also explain the under studied anomaly of enlarged basal ganglia, described for the first time in 5–6 % of MOWS patients in neuroimaging studies [20]. In addition, *ZEB2* downregulation may provide a strategy to attempt to restore striatal mDA innervation, for degeneration of the latter causes major motor dysfunction in Parkinson’s disease [69].

##### Hindbrain

One of the three layers of the cerebellar cortex, i.e., the middle Purkinje cell layer (PCL), contains the soma of Purkinje neurons and Bergmann glia, which interact. Bergmann glia are formed from RGCs between E14.5–E18.5, relocate their soma from the VZ to the PCL, mature around P6, and are required for proper cerebellum layering. These cells are astrocytes that extend their processes into a second layer, the outer molecular layer (OM). The extended processes of Bergmann glia cells serve migration of granule neuron precursors (GNPs, which give rise to the most prevalent neurons in the third layer, the inner granular layer, IGL) in early postnatal stages. 

ZEB2 is present in SOX2+ RGCs in the VZ of the cerebellum at E14.5 and subsequently in Bergmann glia, including at birth and at P6, prompting He et al. (2018) [196] to test whether in the mouse it is required for Bergman glia formation from RGCs, using a panel of Cre-drivers for making *Zeb2*-cKO mice (including *hGFAP*-Cre; *Atoh1*/*Math1*-Cre). *hGFAP*-Cre;*Zeb2*-cKO mice are viable, but develop severe tremors and balance control defects two weeks after birth, similar to *Gsh2*-Cre;*Zeb2*-cKOs (that die between P16-P21) observed by van den Berghe et al. (2013) [64], whereas in heterozygous *Zeb2*-mutant mice this phenotype was not present. The histomorphological and marker analysis of P18-P28 *Zeb-null* mice clearly demonstrate that Zeb2 is required for normal cerebellar development in the mouse. Furthermore, radial migration of GNPs is strongly hampered from P10, due to preceding lower numbers of Bergmann glia already from P3 on, caused by their impaired specification (not increased cell death), defects of which are already visible from E15.5 and peak at E18.5, whereas GNP and Purkinje cell numbers are normal. 

RNA-seq at P0 using cerebella revealed in the Kos upregulated expression of EMT associated genes, tight junction formation, and astrocytic signatures in general, and downregulation of cell cycle and Bergman glia specific genes, including components of the Notch, FGF, BMP pathways. These have previously been shown to regulate Bergman glia development and the Netrin pathway upon their increase. This is a remarkable difference with opposite ZEB2 effects in CNS myelinogenesis and PNS (re)myelination, where ZEB2 generates anti-Notch and anti-BMP effects, again indicating that ZEB2’s actions can be cell-type specific. 

Specifically, although difficult to achieve in cell-type specific manners (see above), the replacement of wild-type ZEB2 with a ZEB2-SBD (subtle) mutant or other (subtle) domain mutants (such as in NIM or CID), should be considered in next-generation approaches for comparing these two different ZEB2 actions in the respective cell types. 

##### Spinal Cord

As a side observation in a study of ONECUT (OC) TFs that act upstream of *Isl1*, encoding a LIM-HD containing TF, in generation of subtypes of OLIG2+ motor neuron (MN) in chick and mouse embryo spinal cord, ZEB2 was identified as a novel marker and developmental regulator of visceral MN differentiation [75]. This positions ZEB2 as a candidate TF that co-regulates the segregation of somatic and visceral MNs at thoracic levels of the spinal cord, innervating each specific target muscles and sympathetic neurons of the paravertebral ganglia, respectively. In *Onecut* cKOs, *Isl1* and *FoxP1* levels remain low and negatively affect the numbers of visceral MNs that are formed, as an expected result. This is paralleled by increased numbers of preganglionic column (PGC) neurons at E12.5 and increased numbers of visceral MNs, which confirms the production of extra PGC neurons, and wherein Zeb2 mRNA and protein are detected. 

This observation was followed up by using *Brn4*/*Pou3f4*-Cre;*Zeb2*-cKO embryos in order to assess whether delayed *Zeb2* expression in *Onecut* mutant mice contributes to the visceral MN phenotype. However, ZEB2 removal results in reduced numbers of PGC MNs, hence ZEB2 may exert effects opposite to OC TFs during visceral MN differentiation, to establish correct numbers of these cells. In addition, stimulation of *Zeb2* by OC TFs might provide an important mechanism to adjust visceral MN production, but indirect evidence points rather at other OC direct target genes and it cannot be excluded thus far that non-cell autonomous effects contribute to the MN subtype defects seen in this study.

#### 5.2.10. *Zeb2*-cKO in Early and Late Retinogenesis, and Lens Formation

MOWS patients present several eye defects, including retinal atrophy and coloboma [14,72], as well as severe myopia for which *ZEB2* was found to be a receptive locus [197]. Furthermore, microphthalmia, chorioretinal and iris coloboma, optic nerve hypoplasia, and cataract are found in MOWS patients. [14,18,72,198,199,200,201]. In *Xenopus* and early mouse embryos, *Zeb2* is expressed in the retina [49,202]. 

The retina is a multi-layered sensorineural epithelium and contains different types of neuron and glial cells over three layers; the photoreceptor layer, the ganglion cell layer (containing retinal ganglion cells which extend into the brain), and the inner nuclear layer (containing interneurons, i.e., horizontal, bipolar and amacrine cells, and Müller glia cells). In a screen for changed genes in Pax6-deficient retinal precursors (RPCs) [203], *Zeb2* was identified and validated as downregulated gene, and its expression in various cell types during mouse retinal development was documented in detail [79]. *α*-Cre;*Zeb2*-cKOs were used to study ZEB2’s role from E12.5 onwards, i.e., in early retinogenesis, including documentation of the phenotype till P14. Striking were the changed ratios between different cell types, grossly increased numbers of Müller glial cells, and reduced numbers of bipolar and amacrine interneurons, while the horizontal interneurons were even completely lost. 

In this study, photoreceptor function as tested by electroretinograms was intact in the *Zeb2*-KOs, but bipolar cell function was lost. Further results show that ZEB2 controls the onset of expression of genes in inner nuclear layer precursors in early retinogenesis. Importantly, *Zeb2* is controlled by PAX6 and directly regulates the level of *Ptf1a*, of which decreased levels are found in the *Zeb2*-cKOs. PTF1a is a most upstream TF of the horizontal/amacrine cell regulatory network, and further analysis by ChIP at E16.5 indicated that this regulation happens via a ZEB2-binding enhancer located 12 kb downstream of *Ptf1a*. Deviating results were reported by another team, where *Zeb2* inactivation in RPCs was reported to result in a primary loss of non-photoreceptor cells, and a cell-fate change from RPCs into photoreceptor cells was claimed to arise from dysregulation of genes involved in photoreceptor differentiation, which are normally repressed by ZEB2 [80]. 

In a follow-up study by the team of R. Ashery-Padan (Tel Aviv, Israel), ZEB2 function was then studied in late stages of retinogenesis, and showed by combining cellular and molecular analyses that ZEB2 inhibits Müller glia cell numbers [86]. RNA-seq revealed that ZEB2 in late retinogenesis in the mouse is needed to inhibit *Hes1* and also (BMP-Smad sensitive) *Id* genes to which ZEB2 binds, and which normally promote Müller glia cell fate and inhibit neural differentiation. Importantly, further analysis in neural progenitors indicate that ZEB2 prevents *Id1* expression through inhibition of BMP-SMAD mediated activation of *Id1*. 

During eye lens formation in the mouse, *Zeb2* expression starts after the induction of the placode, localizes thereafter to the lens epithelium and immature lens fibers. Two different *Pax6*-Cre;*Zeb2*-cKO approaches showed the respective requirement of ZEB2 at two distinct steps, i.e., proper lens vesicle closure and fiber cell differentiation [77]. cKO after the first step of lens vesicle closure showed a dual function for ZEB2 in lens formation: it acts not only during lens vesicle closure (i.e., controlling *FoxE3*, remarkably via proposed ZEB2-Smad9 co-operative activation), but also later during development by activating genes involved in lens development, while repressing ectodermal genes (see [78]). 

The aforementioned studies thus reveal the role of ZEB2 in retinal and lens development, and can help to explain the mechanisms underlying the eye defects in MOWS patients.

#### 5.2.11. *Zeb2*-cKO in Embryonic Myelinogenesis and Postnatal (re)Myelination

To establish proper brain function, myelination is essential and required for optimal conduction velocity of nerve impulses [204], and defects may cause disrupted motor function. Myelination occurs by the formation of myelin sheets generated by oligodendrocytes/Schwann Cells (SCs), which derive from oligodendrocyte precursor cells (OPCs). The differentiation of these OPCs towards mature, myelinating cells is inhibited by the BMP and Wnt, and Notch signaling [205]. 

In addition to early observations that ZEB2 is present in cells that accompany motor neuron axons in the trunk of early mouse embryos at the level of the limbs [8], using whole-genome ChIP-seq the team of R. Lu (Cincinnati, Dallas, TX, USA) had identified ZEB2 as a direct target for the TF OLIG2 via multiple binding sites and, in micro-arrays, as a mRNA that required normal levels of OLIG1. Both TFs are essential for CNS myelinogenesis, and the combination of the results of the two approaches identified ZEB2 as a strong candidate of about 400 OLIG1/2 shared targets in total. Three subsequent key observations were the starting point for functional studies in cells of this somewhat unexpected system. First, analysis of *Olig1* and *Olig2* KO mice revealed that in P14 and E14.5 spinal cords, *Zeb2* mRNA was abnormally downregulated to barely detectable levels. Second, using adult rat hippocampus-derived early OPCs, which can be obtained in reasonable numbers, overproduction of OLIG1 and/or OLIG2 activated *Zeb2*. Third, colocalization with appropriate markers indicated that high levels of ZEB2 were present in mature oligodendrocytes, and only very low levels in OPCs. This prompted the use of an *Olig1*-Cre;*Zeb2*-cKO approach to further demonstrate ZEB2’s requirement in CNS myelinogenesis. These mice are viable, but from postnatal week 2 on they developed generalized tremors, limb paralysis and seizures, reminiscent of other mouse models with defective myelinogenesis and, also in the *Zeb2-null* mice reflected by, e.g., a translucent optic nerve, indicative for, and subsequently shown by microscopy, a severe deficiency in myelination. Further analysis demonstrated that intact ZEB2 is not needed in PDGFRα+ OPCs, but is critical for their differentiation. 

An impressive series of experimental approaches then allowed the following conclusions. As mentioned, ZEB2 downstream of OLIG1/2 is needed during CNS myelinogenesis for differentiation of OPCs to myelinating cells. In this step, its repressor and activator activities are needed. ZEB2 represses BMP-SMAD-activated genes that normally inhibit myelinogenesis, and directly activates *Smad7*, an inhibitory SMAD that in addition to ZEB2 downregulates BMP signaling. However, in a Smurf-dependent manner, Smad7 also sends Wnt-activated β-catenin (β-cat), which also inhibits myelinogenesis, in a degradation pathway. This was a first important observation of cell-intrinsic control of not only BMP-Smad, but also Wnt-βcat signaling by ZEB2. Several resulting predictions could then be tested further, and two were clearly confirmed experimentally. The first is that in the used neonatal rat OPCs and in the presence of PDGF-AA mitogen, ZEB2 overexpression promotes differentiation into even mature oligodendrocytes. As a matter of fact, this OE also results in the downregulation of steady-state levels of transcripts for negative regulators of differentiation, i.e., *Id2* and *Id4*, *Hes1* and *Hes5*, and *Bmpr1a*, and in a strong increase in, e.g., *Sox10* and *Olig2*. Furthermore, other experiments suggest that ZEB2-P300 co-operation is responsible for direct activation of pro-myelination genes. The second is that *Smad7* OE rescued the myelination defect in *Zeb2*-KO OPCs. The third one would be that in the (ubiquitous) *Smad7*-KO mouse, a myelinogenesis phenotype may be present, but to our knowledge this remains to be shown. 

These results encouraged two large consortia in the field to continue to address ZEB2 function in SCs of the PNS, where it is present, e.g., in the SC-marker SOX10+ cells at P7, is high in the first 2 weeks after birth, and then gradually declines till up to 10 weeks [68]. Furthermore, 90% of SCs in sciatic nerves in E18.5 mouse embryos are ZEB2+, decreasing to 70% at P10, to eventually become ZEB2-negative in adult SCs; however, acute sciatic nerve injury already rapidly increases *Zeb2* expression (6 hours after injury) to reach 80% of the cells 7 days after injury, and maintain during remyelination ZEB2+ SCs 2 weeks after injury [67]. These parallel studies turned out exemplary again and led to important confirmations and conclusions. In general, the cellular and molecular phenotypes studied in CNS myelinogenesis (see above) and PNS SC myelination are comparable. However, first, the teams followed grossly two different approaches to document ZEB2’s requirement in controlling signaling pathways that impact SCs, i.e., Notch in this case (see below), by genetic and drug-interference approaches in mice, respectively, rescuing (e.g., the too high Notch-HEY2 related) phenotypes. Second, they highlight ZEB2’s requirement in establishing anti-Notch and anti-SOX2 activity in the ZEB2+ cells, besides the already known anti-BMP and anti-Wnt activities (from CNS myelinogenesis, see above). Third, they also started for the first time to functionally address ZEB2 domain function (i.e., the need for NIM, for interacting with NuRD, using the NIM-deficient ZEB2^R22G^ mutant). Fourth, using inducible Cre-approaches they started to encompass the process of postnatal (re)myelination and demonstrate that *Zeb2*-KO SCs fail to efficiently support regeneration. 

These results in the mouse models recapitulate the phenotypes observed in MOWS patients presenting a delay in myelination and defects in white matter, proposed to cause motor deficiencies such as a delay in walking. 

## 6. Emerging Cellular Models for Studying ZEB2

It will not take long before MOWS patient inspired, subtle protein-coding and likely also enhancer variants/mutations will be introduced at cellular level, i.e., ESCs and iPSCs. The same holds for structure-function studies of ZEB2 that address domain-specific functions to study ZEB2-controlled cell neural (including different subtypes of neuron) as well as general differentiation (providing the possibility to study ZEB2 in TGFβ family ligand stimulated cells). 

These studies can be done in 2D culture and in embryoid bodies, but emerging options are spheroids and organoids. Furthermore, these can also be designed as fused spheroids and organoids, addressing cell–cell communication in novel ways (for example for studies of brain cortex pyramidal neuron–cortical interneuron communication). Hence, it can be predicted that the numbers of reports on ZEB action in these systems will increase. However, in all these systems, the question will be whether in addition to the cell-autonomous actions of ZEB2, also the cell non-autonomous actions will be faithfully recapitulated. 

## 7. General Conclusion and Future Perspectives

ZEB2 research is now using new technologies ranging from multi-omics, even at the single-cell level, to CRISPR/Cas-based tagging and editing, to further study its functions, action mechanisms, partner proteins and target genes. This research will at the same time take the field beyond the originally reported embryonic functions and/or aspects of MOWS congenital malformations. In this respect, the emerging new results on cancer (e.g., melanoma), hematopoiesis (including leukemia) and immune system (where we anticipate its actions will be broader than those thus far reported), are illustrative. Research in the mouse also has told the field that genetic background is important, and can determine the manifestation or penetrance of the defects arising from *ZEB2* mutations, the latter also likely in the future including important enhancers or dysregulation of the expression regulation of the *ZEB2* locus in chromatin context. In addition, more attention should be paid to sometimes cell-type specific miR-based control of ZEB2, and the function of its naturally encoded antisense-transcript in its locus and other lncRNAs.

Of interest are also the novel findings that link Zeb2 to skeletal myogenesis [206] and to the regenerative and protective roles of Zeb2 in the infarcted heart [81] and in liver fibrosis [82], which we do not cover in this review but might provide ground for a future overview.

Moreover, in humans, mutations in the *ZEB2* protein-coding sequence mainly affect the amount of functional, wild-type ZEB2 available within the cells, but fine-controlled Zeb2 levels in knock-out mouse models cannot be easily achieved and may still not precisely mimic the clinical phenotype observed in MOWS patients. Another complication is due to the fact that very few patients carry the same mutations and, in most of these cases, the mutation results in a complete absence of ZEB2 produced from the mutant allele. In addition, MOWS patients without a mutation in the protein-coding sequence of ZEB2 start to be identified, increasing the difficulties in capturing such mutations in mouse models. 

Therefore, new model systems, such as patient-derived induced pluripotent stem cells (iPSCs) as described by Schuster and colleagues [207] will be necessary to fully characterize patient specific phenotypes and causal cellular deficiencies. With the help of such models, a general MOWS gene signature could be annotated, based on time and tissue-specific deregulated genes caused by different *ZEB2* mutations, on documenting whether these genes are direct ZEB2 targets, and on how they depend on an intact functional domain or ZEB2 protein–partner protein interaction site. 

## Figures and Tables

**Table 1:** Mouse models associated with MOWS-like phenotypes (with Δ = the result of Cre action).

Model	Phenotype Underlying MOWS-Like Defects	Publication
*SIP1* *fl* *ox(ex7)* *SIP1* *fl* *ox(ex7)* *EIIa-Cre; Zeb2* ^Δ*ex7/*Δ*ex7*^	Early post-gastrulation embryonic lethality, failure of neural tube closure, cranial NCC delamination and migration, and vagal NCC generation, defected somite boundary positioning	Higashi et al., 2002, van de Putte et al., 2003, Maruhashi et al., 2005 [48,53,57]
*Zp3-Cre; Zeb2^+/^*^Δ^*^ex7; ^*Δ*EF1^+/−^*	Defect in somite production and developmental arrest at E8.5, severe defected dorsal neural tube	Miyoshi et al., 2006 [58]
*Emx1-Cre^+/−^; Zeb2^KO/^* ^Δ*ex7*^	Lack of hippocampus and corpus callosum	Miquelajauregui et al., 2007 [61]
*Wnt1-Cre^+/−^; Zeb2^KO/^* ^Δ*ex7*^	Abnormal craniofacial, hearth and melanocyte development and defects in the PNS of the gastrointestinal tract and sympatho-adrenal lineage	van de Putte et al., 2007 [8]
*Nestin-Cre^+/−^; Zeb2^KO/^* ^Δ*ex7*^	Defects in cortical layering and in interneuron migration	Seuntjens et al., 2009 [47]
*Nex-Cre^+/−^; Zeb2^KO/^* ^Δ*ex7*^	Defects in cortical layering	Seuntjens et al., 2009 [47]
*Nex-Cre^+/−^; Zeb2^KO/^*^Δ*ex7*^; *Ntf3^−/−^*	Defects in cortical layering	Seuntjens et al., 2009 [47]
Olig1-Cre^+/−^; Zeb2^Δex7/Δex7^	Defects in the maturation of precursor cells to oligodendrocytes and impaired myelin formation	Weng et al., 2012 [62]
*Nkx2.1-Cre^+/−^; Zeb2^KO/^* ^Δ*ex7*^	Defects in GABAergic interneuron migration	McKinsey et al., 2013, van den Berghe et al., 2013 [63,64]
*Gsh2-Cre^+/−^; Zeb2^KO/^* ^Δ*ex7*^	Defects in GABAergic interneuron migration and seizures	van den Berghe et al., 2013 [64]
*Dlx5/6-Cre^+/−^; Zeb2^KO/^* ^Δ*ex7*^	Defects in GABAergic interneuron migration	van den Berghe et al., 2013 [64]
Zeb2^Δ^*^ex7/+^*; *pure C57BL/6N*	Craniofacial abnormalities, defective corpus callosum formation, decreased numbers of parvalbumin interneurons in the cortex, reduced motor activity, increased anxiety, and impaired sociability	Takagi et al., 2015 [65]
Nex-Cre^+/−^; Zeb2^+/^*^Δ^^ex7^*	Defects in axonal growth and ipsilateral intracortical collateral formation	Srivatsa et al., 2015 [66]
*Dhh-Cre^+/−^; Zeb2* ^Δ*ex7/*Δ*ex7*^	Arrest of Schwann Cell differentiation during peripheral nerve development and inhibition of remyelination after injury	Wu et al., 2016, Quintes et al., 2016 [67,68]
Dhh-Cre; Zeb2*^Δex7/Δex7^*; Ednrb*^Δ^^/^^Δ^* Dhh-Cre; Zeb2*^Δ^^ex7/^^Δ^^ex7^*; Hey2*^Δ^^/^^Δ^*	More mature axon-Schwann Cell units	Quintes et al., 2016 [67]
*Nestin-Cre^+/−^; Zeb2^KO/^* ^Δ*ex7*^	Increased BMP/Smad dependent axon growth and dopaminergic hyperinnervation in the striatum	Hegarty et al., 2017 [69]
*Gsh2-Cre^+/−^; Zeb2^KO/^* ^Δ*ex7*^	Defects in differentiation and maturation of olfactory bulb interneurons	Deryckere et al., 2020 [30]
Gfap-Cre^ERT2^; *Zeb2*^Δ*ex7/*Δ*ex7*^	Larger lesions, and delays recovery of motor function after spinal cord injury or ischemic stroke	Vivinetto et al., 2020 [70]
*Nex-Cre; Zeb2* ^Δ*ex7/*Δ*ex7*^	Decreased expression of excitatory receptors and an impaired Ca^2+^ signaling	Turovskaya et al., 2020 [71]
*Zeb2^+/−^; Sox10^+/−^*	Defects in ENS	Stanchina et al., 2010 [59]
*Zeb2^KO/+^; Ednrb^s^* *Zeb2^KO/+^; Edn3^ls^*	Severe enteric anomalies and increased neuronal differentiation	Watanabe et al., 2017 [72]
*Zeb2^+/KO^*	Reduced pain response, defects in nociceptive transduction signals	Jeub et al., 2011 [73]
*Zeb2^+/KO^*	Reduced pain response, defects in DRG neuron development	Pradier et al., 2013 [74]
*Brn4-Cre^+/−^; Zeb2^KO/^* ^Δ*ex7*^	Defects in visceral motor neurons	Roy et al., 2012 [75]
*Tyr-Cre; Zeb2* ^Δ*ex7/*Δ*ex7*^	Defects in melanoblast migration and melanocyte differentiation	Denecker et al., 2014 [76]
*Pax6(Lens)-Cre;* *Ze* ^Δ*ex7/*Δ*ex7*^ *Pax6(LP)-Cre;* *Zeb2* ^Δ*ex7/*Δ*ex7*^	Defects in vesicle lens closure and defects in lens fiber maturation	Yoshimoto et al., 2005 [77]
*MLR10-Cre; Zeb2* ^Δ*ex7/*Δ*ex7*^	Defects in coordinated cell migration, cataract formation and abnormalities in fiber cell organization in the lens	Manthey et al., 2014 [78]
α-Cre; Zeb2^Δ*ex7/*Δ*ex7*^	Defects in cell numbers of various neuronal and glial cell types in the retina	Menuchin-Lasowski et al., 2016 [79]
*Six3-Cre; Zeb2* ^Δ*ex7/*Δ*ex7*^	Loss of non-photoreceptor cells, switch in cell fate to photoreceptor cells by retinal progenitors and increased apoptosis	Wei et al., 2019 [80]
*Nrc1^iCre;^ Zeb2* ^Δ*ex7/*Δ*ex7*^	Impaired NK cell maturation, survival and bone marrow exit	Van Helden et al., 2015
*Nrc1^iCre;^ R26-Zeb2^Tg^*	Decreased NK cells in the bone marrow and an increase in mature NK cells in the spleen and bone marrow	Van Helden et al., 2015 [39]
*αMHC-Cre; Zeb2* ^Δ*ex7/*Δ*ex7*^	Impaired cardiac contractility and infarct healing post-myocardial infarction	Gladka et al., 2021 [81]
αMHC-Cre-R26Zeb2*^OE^*	Improved cardiomyocyte survival and cardiac function	Gladka et al., 2021 [81]
Cdh5-Cre^ERT2^; *Zeb2*^Δ*ex7/*Δ*ex7*^	Expanded liver vasculature and irregularities in the angioarchitecture	De Haan et al., 2021 [82]
Cdh5-Cre^ERT2^; R26-Zeb2^OE^	Reduced vascularity and attenuated CCl_4_-induced liver fibrosis	De Haan et al., 2021 [82]
*Tyr-Cre^ERT2^; Zeb2* ^Δ*ex7/*Δ*ex*^ *and Tyr-NRAS ^p53^*	Decreased outgrowth of primary melanomas	Bruneel et al., 2020 [83]
*R26-Zeb2^OE/OE^iresGFP*	Increased proliferation and growth of primary and secondary melanomas	Bruneel et al., 2020 [83]

**Table 2:** Identified *ZEB2* enhancers.

Model System	Location	Activity	References
*Rat*	rChr3: 26822763-26823523	Post-natal kidney development	El-Kasti et al., 2012 [121]
*Mouse*	chr2:43,978,103-43,978,294	GABA-ergic interneurons in developing subpallium	McKinsey et al., 2013 [63]
*Zebrafish*	Zeb2#e2: intron, Chr2, 14518542-14518630	Notochord	Bar-Yaacov et al., 2019 [122]
*Zebrafish*	Zeb2#e3: intron, Chr2, 145188070-145189835	Mid/hindbrain, spinal cord, forebrain	Bar-Yaacov et al., 2019 [122]
*Zebrafish*	Zeb2#e4: intron: Chr2, 145196296-145197640	Notochord, non-specific neurons	Bar-Yaacov et al., 2019 [122]
*Zebrafish*	Zeb2#e5: intron, Chr2, 145201196-145202221	Mid/hindbrain, somatic muscles, spinal cord	Bar-Yaacov et al., 2019 [122]
*Zebrafish*	Zeb2#e6: intron, Chr2, 145209727-145210776	Trigeminal-like ganglia, somatic muscles	Bar-Yaacov et al., 2019 [122]
*Zebrafish*	Zeb2#e7: intron, Chr2, 145215740-145216978	Trigeminal-like ganglia	Bar-Yaacov et al., 2019 [122]
*Zebrafish*	Zeb2#e12: intron, Chr2, 145265457-145266567	Notochord	Bar-Yaacov et al., 2019 [122]
*Zebrafish*	Zeb2#e13: intron, Chr2, 145267933-145268902	Somatic muscles	Bar-Yaacov et al., 2019 [122]
*Zebrafish*	Zeb2#e14: intron: Chr2, 145272461-145274126	CNS	Bar-Yaacov et al., 2019 [122]
*Human iPSCs*	E1: Chr2:145764483–145765504	NPC differentiation	Birkhoff et al., 2020 [123]
*Human iPSCs*	E2: Chr2:145769677–145770210	NPC differentiation	Birkhoff et al., 2020 [123]
*Human iPSCs*	E3: Chr2:145779965– 145780193	NPC differentiation	Birkhoff et al., 2020 [123]

**Table 3:** ZEB2 associated miRNAs.

miRNA	Regulation	References
*miRNA-192*	TGFβ-induced collagen expression, and diabetic kidney glomeruli	Kato et al., 2007 [132]
*miRNA-200 family*	EMT	Bracken et al., 2008; Christoffersen et al., 2007; Gregory et al., 2008; Perdigão-Henriques et al., 2016 [125,127,129,130]
	EMT and cancer cell migration	Korpal et al., 2008 [126]
	Epithelial phenotype of cancer cells	Park et al., 2008 [136]
*miRNA-200*	EMT in iPSCs	Wang et al., 2013 [128]
*miRNA-205*	Renal carcinoma	Chen et al., 2014 [137]
*miRNA-9*	Rat brain cortical development	Kropivsek et al., 2014 [131]
*miRNA-145*	EMT and stem cell properties in prostate cancer	Ren et al., 2014 [135]
*miRNA-200 family*	Post-natal forebrain neurogenesis	Beclin et al., 2016 [124]
*miRNA-215*	Metastasis of colorectal cancer	Chen et al., 2017 [138]
*miRNA-30a*	Triple negative breast cancer aggressiveness	Di Gennaro et al., 2018 [139]
*miR200 family*	CD8+ cell fates	Guan et al., 2018 [140]
*miRNA-200b*	Migration and invasion of oral squamous cell carcinoma	Ren et al., 2018 [141]
*miRNA-203*	Colon cancer liver metastasis	Wang et al., 2018a [142]
*miRNA-146*	Expression and replication of Hepatitis B virus	Wang et al., 2018b [143]
*miRNA-206*	Proliferation of renal clear cell carcinoma	Chen et al., 2019a [144]
*miRNA-30a*	Human nasopharyngeal carcinoma	Chen et al., 2019b [145]
*miRNA-505*	Metastasis and EMT in cervical cancer	Feng et al., 2019 [146]
*miRNA-1179*	Metastasis of hepatocellular carcinoma	Gao et al., 2019 [147]
*miRNA-101*	Proliferation and invasion of osteosarcoma	Lin et al., 2019 [148]
*miRNA130b-3p*	Migration and invasion of non-small lung carcinoma	Qu et al., 2019 [149]
*miRNA-940*	EMT in glioma cells	Xu et al., 2019 [150]
*miRNA-155*	Migration and invasion of colorectal cancer cells	Yang et al., 2019a [133]
*miRNA-145*	Senescence of activated hepatic stellate cells	Yang et al., 2019b [134]
*miRNA-498*	Growth and metastasis of liver cancer	Zhang et al., 2019a [151]
*miRNA-200c-3p*	Tumor progression of prostate carcinoma	Zhang et al., 2019b [152]
*miR-124, miRNA-203*	EMT renal carcinoma	Chen et al., 2020 [153]
*miRNA-215-5p*	EMT in podocytes	Jin et al., 2020 [154]
*miRNA-200a*	Proliferation in drug-induced gingival overgrowth	Lin et al., 2020 [155]
*miRNA-200c-3p*	Proliferation and migration of renal artery endothelial cells	Liu et al., 2020 [156]
*miRNA-200b*	Senescence and inflammatory responses in pulmonary emphysema	Shen et al., 2020 [157]
*miRNA-138*	Progression of colorectal cancer	Yan et al., 2020 [158]
*miRNA-140*	Progression of esophageal cancer	Yang et al., 2021 [159]

## Data Availability

Not applicable.
